# Development and Comparison of Two Assay Formats for Parallel Detection of Four Biothreat Pathogens by Using Suspension Microarrays

**DOI:** 10.1371/journal.pone.0031958

**Published:** 2012-02-15

**Authors:** Ingmar Janse, Jasper M. Bok, Raditijo A. Hamidjaja, Hennie M. Hodemaekers, Bart J. van Rotterdam

**Affiliations:** 1 Laboratory for Zoonoses and Environmental Microbiology, National Institute for Public Health and the Environment (RIVM), Bilthoven, The Netherlands; 2 Laboratory for Toxicology, Pathology, and Genetics, National Institute for Public Health and the Environment (RIVM), Bilthoven, The Netherlands; Naval Research Laboratory, United States of America

## Abstract

Microarrays provide a powerful analytical tool for the simultaneous detection of multiple pathogens. We developed diagnostic suspension microarrays for sensitive and specific detection of the biothreat pathogens *Bacillus anthracis*, *Yersinia pestis*, *Francisella tularensis* and *Coxiella burnetii*. Two assay chemistries for amplification and labeling were developed, one method using direct hybridization and the other using target-specific primer extension, combined with hybridization to universal arrays. Asymmetric PCR products for both assay chemistries were produced by using a multiplex asymmetric PCR amplifying 16 DNA signatures (16-plex). The performances of both assay chemistries were compared and their advantages and disadvantages are discussed. The developed microarrays detected multiple signature sequences and an internal control which made it possible to confidently identify the targeted pathogens and assess their virulence potential. The microarrays were highly specific and detected various strains of the targeted pathogens. Detection limits for the different pathogen signatures were similar or slightly higher compared to real-time PCR. Probit analysis showed that even a few genomic copies could be detected with 95% confidence. The microarrays detected DNA from different pathogens mixed in different ratios and from spiked or naturally contaminated samples. The assays that were developed have a potential for application in surveillance and diagnostics.

## Introduction

A number of pathogens are considered to be a potential threat to public health, even though their incidence is low under normal circumstances. Of major concern is a deliberate release of such biothreat micro-organisms, which could enhance their spread and exposure and could result in their occurrence in unanticipated locations and environments. At least 30 highly pathogenic micro-organisms, which are very diverse [1], can be considered biothreat agents. Timely recognition of disease agents will enable appropriate treatment of exposed individuals which is critical to their survival, and spread of disease can be reduced by taking public health measures. Thus, reduction of the impact of these pathogens demands accurate and rapid diagnostic tools for their detection. False-positive and false-negative test results must be prevented because they might cause unnecessary or missed responses (and a lost opportunity for intervention), which are unacceptable when dealing with deadly biological threats. To minimize false-positive and false-negative measurements, the detection methods should be specific, sensitive, cover multiple pathogens and multiple targets for each pathogen, and include appropriate controls. To achieve this, it is highly beneficial to analyze multiple analytes simultaneously, which will reduce sample handling, sample consumption and time required for the analysis. Such multiplex techniques for the detection of dangerous pathogens will enable the screening of diverse suspect samples. In addition, these techniques could be valuable for rapid diagnostics of human and animal samples, especially in cases when clinical symptoms are not differentiating.

Culture-based methods for pathogen identification are the gold standard and can be highly sensitive. Nevertheless, such methods are not adequate for rapid detection, because they are time-consuming, not always very specific, involve extensive biosafety measures, and some organisms simply resist cultivation [2]. Molecular methods for the detection of pathogens can be equally or more sensitive and can provide higher speed and specificity. Also, such methods require only preparatory handling of samples under biosafety conditions and can be easily scaled-up, which is important for speeding up investigations and control of disease progression in outbreak situations. Real-time PCR (qPCR) offers exquisite sensitivity, specificity and speed. However, multiplexing capabilities are limited (to maximally 5 targets), while significant multiplexing is needed for the diagnosis of multiple pathogens and to ascertain reliable detection by inclusion of redundant targets and internal controls. Required multiplexing capabilities can be realized by using microarrays. Microarrays resolve complex mixtures of amplified products and are thus very suitable for parallel detection of multiple targets. However, to achieve sensitive detection, target DNA needs to be amplified before microarray hybridization. For that reason, the trade-off for the application of diagnostic microarrays is an increase in overall assay time compared to qPCR.

Application of Luminex® xMap technology for the construction of diagnostic microarrays offers several advantages. This microarray format constitutes of a suspension of small, color-encoded beads conjugated with probes. Such suspension microarrays exhibit rapid hybridization kinetics, flexibility in assay design and low cost [3]. Microarrays can be compiled as desired by adding or replacing beads (and probes) without having to reformat and print new arrays (a disadvantage of planar microarrays). Diagnostic microarrays for the detection of multiple pathogens using xMap technology have been developed and assays for respiratory pathogens have been commercialized (Resplex I and II from Qiagen, MultiCode-PLx RVP from Eragen Biosciences and xTAG™ RVP from Luminex Molecular Diagnostics) [4], [5]. Application in biodefense screening has been explored by Wilson *et al.* [6] who described an assay for the detection of 4 biothreat bacteria, without disclosing oligonucleotide sequences.

Before signature sequences can be detected by measuring hybridization to the microarrays, pathogen DNA needs to be amplified and labeled. There are two different strategies to accomplish amplification, labeling and hybridization. In one format (direct hybridization, DH), labeled primers are used in multiplex PCR to generate PCR products that can be detected and discriminated using template-specific conjugated microarray probes. An alternative format (xTAG) uses multiplex PCR in the first step, followed by a sequence-specific enzymatic step which incorporates the label and a unique capture (TAG) sequence. Detection occurs by hybridization to (anti-TAG) probes on a universal microarray. If the enzymatic reaction for sequence discrimination is strand extension by DNA polymerase, the assay chemistry is called target-specific primer extension followed by universal hybridization (TSPE-UH). Each assay format has its specific advantages and shortcomings which could influence its performance. The xTAG format is claimed to be more specific, allowing detection of single nucleotide polymorphisms (SNPs), due to the additional selective primer extension (or ligase) step combined with the optimized universal array hybridization [3]. On the other hand, the direct hybridization format requires a simpler and more rapid procedure due to the lack of an additional, time-consuming enzymatic reaction, and to the availability of convenient magnetic beads. Although high specificity can be achieved by performing careful probe design, differentiation of SNPs based solely on hybridization may be more difficult. Both DH [6], [7], [8], [9] and TSPE-UH formats [10], [11] have been used for multiplex detection of pathogens. While setting up a suspension microarray for the detection of bacterial pathogens, we were confronted with a lack of studies directly comparing the performance of different assay chemistries. Significant performance features not only include specificity, sensitivity and speed, but also the robustness of the data that are generated, for instance the signal to background ratio of positive signals. The aim of our study was to develop and validate suspension microarrays for the detection of biothreat pathogens, and investigate the effect of the assay chemistries DH and TSPE-UH on microarray performance.

## Results

### Oligonucleotide design and multiplex (asymmetric) PCR amplification

Microarrays were designed for the simultaneous detection of 4 important biothreat pathogens: *B. anthracis*, *Y. pestis*, *F. tularensis* and *C. burnetii*. The first three bacteria are listed as Category A biothreat pathogens while C. burnetii is a Category B pathogen (classification of the CDC, USA, http://www.bt.cdc.gov/agent/agentlist-category.asp) because of the potential danger of their deliberate release. Three or four signature sequences were selected for each organism to ensure sensitive and reliable detection and to provide additional information about virulence and genotypes. Inclusion of a signature sequence from *Bacillus thuringiensis* enabled the use of its highly refractory spores as a control for both DNA extraction and microarray detection, which is especially useful for environmental samples.

**Figure 1:**
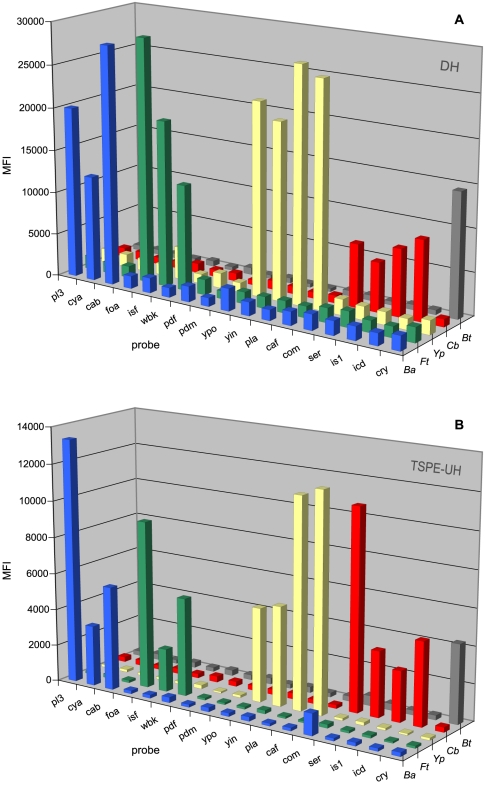
Typical results from DH and TSPE-UH suspension microarrays detecting select pathogens. Two 17-plex bead arrays were developed for the detection of *B. anthracis* (Ba), *F. tularensis* (Ft), *Y. pestis* (Yp), *C. burnetii* (Cb) and an internal control for DNA extraction and microarray detection (Bt). The microarrays were based on (**A**) direct hybridization (DH), or (**B**) target specific primer extension combined with universal microarray hybridization (TSPE-UH) assay formats. Both microarrays make use of identical amplification products from a 16-plex asymmetric PCR. Mean fluorescence intensity (MFI) is displayed for the different probes that are given in Table 1.

Our aim was to design two different suspension microarrays which would be suitable for comparing the performance of the assay chemistries direct hybridization (DH) and target-specific primer extension plus xTAG universal hybridization (TSPE-UH). To enable a direct comparison between the two formats, 17 oligonucleotides were designed for optimal hybridization at 55°C and which could function as hybridization probe for DH or as TAG-ged TSPE primer (Table 1). The 5′-end of these oligonucleotides were coupled to magnetic beads (DH) or extended with a unique TAG sequence (TSPE-UH). Subsequently, primersets were designed for multiplex amplification (and labeling in the case of DH) of 150–300 bp sequences spanning the probe regions while leaving at least 50 bp for TSPE extension (Table 1). Multiplex PCR using pathogen genomic DNA showed products of the expected sizes on gels, although some could not be differentiated due to their similar sizes (data not shown). Protocols for the detection of these PCR products by using DH and TSPE-UH microarrays were optimized by varying the application of washing steps, the amount of TSPE primers (TSPE-UH), and the amount of PCR product used for hybridization (DH). In a first series of experiments, TSPE microarrays performed well, but the DH microarrays showed two major drawbacks. Firstly, the signal to background ratio was low, which made it difficult to confidently recognize hybridization signals in each measurement. Secondly, the signal decreased significantly when higher loads of PCR products were hybridized, which necessitated for each sample an estimation of the optimal load of PCR products for hybridization from a dilution series of PCR products. Both phenomena could be indicative for competition between re-hybridization of PCR products to fixed probes and hybridization to complementary strands. Therefore, we redesigned the multiplex PCR to produce predominantly single stranded PCR products. The unlabeled primers were redesigned according to the LATE PCR requirements [12], [13]. Multiplex asymmetric PCR amplification was optimized by varying primer concentrations, thermocycling times and number of cycles. Successful amplification was monitored by the visualization of double-stranded PCR products on gels, but as before, amplification could not be confirmed for all amplicons due to their similar sizes (data not shown). The labeled, single stranded PCR products were used for both microarray formats. A typical readout of both microarray formats for the detection of the targeted pathogens is given in Fig. 1. Signals from matching probes were very distinct in both methods, but TSPE measurements displayed lower and less variable background signals when compared to DH.

**Table 1:** Oligonucleotides used for amplification and labeling of signature sequences and as fixed probes.

Target organism	Signature sequence	Primer	Primer sequence (5′- 3′)	Exc/Lim^a^	Amplicon size	Probe/TSPE primer	Probe sequence (5′- 3′)
*Bacillus anthracis*	pl3	pl3_f	AGCTTTTTCTCTTGATAGTTTACTAGTTAC	L	243	pl3	CTCGTTCGTGTGTTGAATTAATTACAA
		pl3_r	ACCATGTGTTTCTTTAAGGAATAAC	X			
	cya	cya_f	CTGTTAATAAGTTAGCTACAAACCTTATTA	X	199	cya	CCATGCTTCTTAGATAAATCTTGATCAAA
		cya_r	CCTTCATGCTCTGTAATTGATTTTTTATTTT	L			
	capB	cab_f	CTTTCACTGCTACCATTCCATA	X	294	cab	CCAAGTATTCACTTTCAATAGTGACTAA
		cab_r	ATTGGTCAGCAAAACGTGTAATTC	L			
*Yersinia pestis*	ypo393	ypo_f	CAATGCGCTTTATCCTTTAGTGTATAT	L	282	ypo	AATTTAGTTAATAGCCTTAAGAAATTAAAT
		ypo_r	GTGCCCACCTTCAATTC	X			
	yihN	yin_f	TCGGCCATAGCGATGATCTTATT	L	250	yin	CCATGATTCGATGAACGTATACT
		yin_r	CCTCAAAATTATCTAATAATGAGCCATAAA	X			
	pla	pla_f	AGTGGACAGATCACTCATCTCA	L	213	pla	CAGGATGAGAATTATAAAGCAGGTATAACA
		pla_r	CCCGCACTCCTTTCG	X			
	caf1	caf_f	CCCGCATCACTCTTACATATAA	X	276	caf	CCAACAAGTAATTCTGTATCGATGTTTC
		caf_r	CCACAAGGTTCTCACCGTTTAC	L			
*Francisella tularensis*	fopA	foa_f	CGCTGCAGGTTCAGATAATATC	L	222	foa	GCAGTGGTTTTGCAGCTAATAATTT
		foa_r	GCACCTGATGGAGAGTTAG	X			
	wbtK	wbk_f	CAAGCAAACCTACTATGTTGTATATTACC	L	256	wbk	ATGAAAAACTCCCGTACATCTTG
		wbk_r	ATCAAAAAAAGTATCCGGATATCA	X			
	IS*Ftu2*	isf_f	TGTTACGTACAGGCTGTCA	X	268	isf	TGTAAATCAGGGTTTTGTACTGATTTAAA
		isf_r	GCATCAGTCATAGCATGGATTTTAGT	L			
	pdpD	pdp_f	CATCCAAGTTGAGGACCATA	X	307/451^b^	pdf	AAATCCTGCTGAGCAGAATTTTCT
		pdp_r	AGTTTATAAAGCTCTCTCAAAGAACCTAT	L		pdm	AAAATCTAAGTTTTCACCACTAAACAAT
*Coxiella burnetii*	com1	com_f	AGTTTTTCTCCTCAACAAGTCAAA	X	257	com	GTGATGCAGGGTCGTTAAATAAT
		com_r	GCTTTGCAATGGCCACATTGATA	L			
	serS	ser_f	AAGCTTTGCAGGTTGGTCTTAA	L	173	ser	ACACGGTGCAGTCAAAAAAC
		ser_r	TTTACCTTGGGCCACATAAC	X			
	IS1111	is1_f	GCGCTGTTAAAGATACGCGATC	L	212	is1	GTTGTTGCAAGAATACGGACTC
		is1_r	CGGTTCAACAATTCGGTATACA	X			
	icd	icd_f	GACTTACCAACACATCAAAGTTCC	L	302	icd	AAGGTGAAAAAATCACCGTTAATAAAG
		icd_r	CTTTAATGGCCACTTGGTAT	X			
*Bacillus thuringiensis*	cry1	cry_f	CGGTGAATGAGCTGTTTACTTCTTC	L	230	cry	ATCCAATTTAGTTGAGTGTTTATCAGA
		cry_r	GCCACGGTCTAGTTGTCTA	X			

a Excess primer = X, Limiting primer = L.

b *F. tularensis* subspecies *tularensis* yields amplicon of 307 bp, subspecies *novicida* and *mediasiatica* of 451 bp.

### Specificity of DH and TSPE-UH microarrays

The specificity of each microarray probe (functioning either as bead-coupled hybridization probe or as TSPE primer) was investigated by microarray measurements of asymmetric PCR products generated from single target amplicons. These single target amplicons had been produced from genomic DNA and included the region amplified by multiplex asymmetric PCR, extended with at least 50 bp upstream and downstream sequences. The single target amplicons produced a signal from the matching beads only, with the following exceptions. In the DH array, in some measurements the beads carrying *ypo* and *isf* probes showed slight cross-reactivities with targets *cya* and *caf*, respectively. The mean fluorescence intensity (MFI) was mostly 10% of the matching probe signal, in one measurement 30%. The low cross-reactivities in some DH measurements were confirmed in experiments that were carried out to calculate the limit of detection (LOD), which will be described in more detail below. In these LOD experiments, dilutions of target amplicons mixtures and of genomic DNA from *B. anthracis* and *F. tularensis* showed that cross-reactivity only occurred at the highest target concentrations. In the TSPE-UH array, cross-reaction of *pl3* was found with high concentrations of *Y. pestis* target amplicons and genomic DNA (MFI 5–40% of matching probe signals). In addition, the *com* signal from the TSPE-UH microarray showed considerable cross-reactivity with most target amplicons when tested separately (MFI mostly about 25% of the matching probe signal, occasionally approaching 60%). The corresponding LOD experiments revealed that this cross-reactivity occurred randomly and was not correlated to target concentration. Nevertheless, com probes were maintained in the microarrays during validation experiments as they were illustrative for differences between assay chemistries.

**Figure 2:**
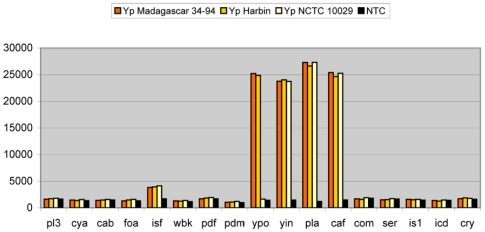
DH suspension microarray measurement showing minor cross-reactivity. Three *Y. pestis* strains (Yp) and the no template control (NTC) are shown from a DH measurement. Probe *isf* showed a minor signal when a high load of *Y. pestis* genomic DNA was amplified.

A panel of organisms was used to validate specificity and strain coverage of the microarrays (Table S2). This panel included DNA from different strains of the targeted pathogens, from closely related Bacteria, and a selection of non-related Bacteria and Eukarya. Genomic DNA measurements showed for both assay formats a very clear difference between matched probe signal and background for most probes (Fig. 1). Nevertheless, in some DH microarray measurements, the signal-to-noise ratio was less pronounced. Moreover, probes targeting *isf* and *ypo* showed slight cross-reactivities with DNA from *Y. pestis* and *B. anthracis*, respectively. Fig. 2 shows data from an experiment in which this effect was measured most prominently. These cross-reactivities were not consistently measured and only in the presence of a high load of the cross-reacting pathogen. These findings were congruent with the findings of single target amplicons (above). This was also true for the TSPE measurements of genomic DNA which confirmed cross-reactivities of the *com* probe and slight cross-reactivity of *pl3* probes.

To deal with the variation between experiments with regard to background MFI, signal-to noise ratios, and the occurrence of cross-reactivities, we formulated a rule for scoring positives. This was required to decide between hybridized probes and background signals in all experiments that were performed to assess microarray performance. By using for each probe a cutoff of 3× the average MFI, calculated from the no template controls (NTC) from all experiments carried out during the study period, hybridization signals could be correctly identified in the DH measurements. For TSPE-UH data, a cut-off of 5 times the average of the NTC probes was used to correctly score positive samples. It should be stressed that the signals from intended hybridizations are well above this cutoff (Figs. 1 and 2). Less stringent rules resulted in positive signals from some of the weakly cross-reacting probes described above, however, this would not lead to misidentifications as complementary pathogen signatures were absent.

Application of the scoring rules to microarray measurements of the DNA panel confirmed the detection of all strains of the targeted pathogens (Table S2). Additional strain information could be derived from the hybridization patterns of the diagnostic signatures. The hybridization patterns of the oligonucleotides designed for signature recognition were as expected (see materials and methods) and were congruent with qPCR results [14]. The only major exception was the *com* primer of the TSPE-UH array, which reacted aspecifically with multiple DNA samples. This was consistent with the observations presented above and disqualifies its use for the detection of *C. burnetii*. In contrast, when used as a probe in the DH array, this oligonucleotide did not show any cross-reactivity and could be used with confidence.

### Sensitivity of DH and TSPE-UH microarrays

Dilutions of target amplicons were used to calculate for each bead and for both microarray formats the Limit of Detection (LOD). Probit regression analysis was used for LOD calculations. LODs for single targets ranged from 12 to 284 copies per reaction. Although the LODs were largely in the same range for both assay formats, for some signatures they were lower when using the TSPE-UH microarray format (for SerS more than 5 times).

LODs for genomic DNA were calculated from dilutions of bacterial DNA extractions (Table 2). For *C. burnetii*, LODs could not be calculated due to impurity of the genomic materials (which contain host DNA). LODs obtained from both microarray formats were very similar. Based on the most sensitive signatures for each pathogen, LODs were 29 fg for *B. anthracis*, 5 fg for *F. tularensis* and 28 fg for *Y. pestis* when using the TSPE-UH microarrays. For DH microarrays, the LOD for *B. anthracis* gDNA was slightly higher (44 fg).

**Table 2:** Detection limits of the DH and TSPE-UH suspension array formats.

	Signature sequence	LOD target amplicons (copies/reaction)^a^		LOD gDNA (fg/reaction)^a^	
organism		DH	TSPE-UH	DH	TSPE-UH
*B. anthracis*	pl3	21	21	44	33
	cya	53	18	58	29
	capB	17	17	64	36
*F. tularensis*	FopA	156	156	157	172
	ISFtu2	12	12	6	5
	wbtK	284	117	704	1093
	pdpD f	128	131	ND	ND
	pdpD m	65	49	ND	ND
*Y. pestis*	YP00393	197	103	752	683
	yihN	63	63	1414	1358
	pla	48	48	28	28
	caf1	12	12	259	259
*C. burnetii*	com1	156	62	ND	ND
	serS	91	17	ND	ND
	IS1111	13	13	ND	ND
	icd	209	151	ND	ND

a Values displayed represent the lowest DNA concentration at which 95% of the positive samples are detected, as calculated by using probit analysis. ND = not determined.

### Mixed pathogens detection

**Figure 3:**
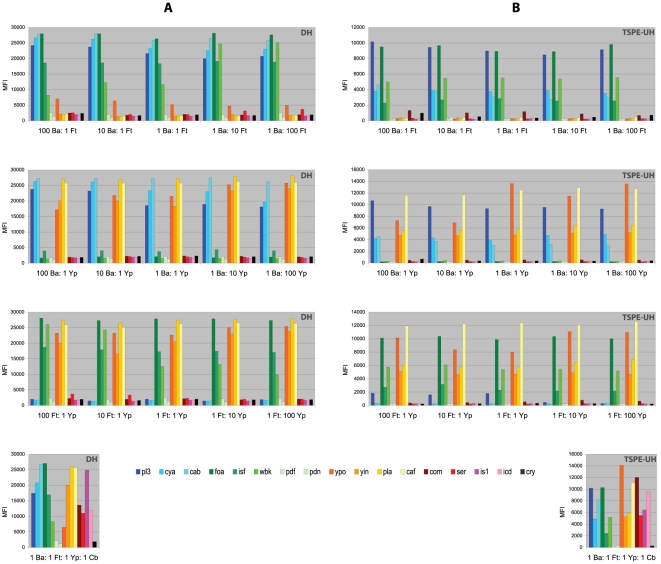
Detection of mixed pathogens by using DH and TSPE-UH suspension microarrays. Genomic DNA from *B. anthracis* (Ba), *F. tularensis* (Ft), *Y. pestis* (Yp), *C. burnetii* (Cb) was mixed in different ratios and measured by using DH (**A**) and TSPE-UH (**B**) microarrays. Mean fluorescence intensity (MFI) is displayed for the different pathogen-specific probes (Table 1). The detection of one pathogen is not impeded by the detection of the other targeted pathogens.

A major benefit of using microarrays is the option to detect multiple pathogens simultaneously. The capability of the developed microarrays to detect pathogens even if other targeted pathogens are present in excess, was investigated by using pathogen DNA mixed in various ratios. Both DH and TSPE-UH microarrays detected mixed pathogens, including if they were all present (Fig. 3). Also, mixtures of two pathogens could be detected when present in different ratios. The MFIs of the various probes appeared to be unaffected by the additional amplifications. Nevertheless, in DH measurements there seemed to be two exceptions. The *wbk* signal was relatively weak at low *Francisella* target input, and in the DNA mixture of all 4 pathogens, the *ypo* signal was relatively low (Fig. 3). Fig. 3 also confirms the finding that low signals from the *ypo* and *isf* probes in the DH microarrays should be interpreted with care, because of the signals originating from slight cross-reactivity with strong signals from *cya* and *caf*, respectively.

### Detection in diverse sample matrices

Our experiments assessed the analytical performance of the developed diagnostic microarrays. Application of these microarrays for multiplex pathogen detection in realistic samples requires recognition of pathogen DNA extracted from complex samples of diverse origins. To investigate whether the methods we routinely use in our laboratory for DNA extraction from complex samples would yield DNA preparations suitable for microarray detection, we measured a few representative veterinary and environmental real-life samples. For this purpose, DNA extraction was performed on samples from goat blood and feces, surface water concentrate and coffee creamer and these extracts were spiked with *Y. pestis* DNA. In addition, DNA was extracted from a surface area swab and from a vaginal swab collected on a goat farm that was known to be contaminated with *C. burnetii*. All samples were spiked with *B. thuringiensis* spores that served as positive controls for DNA extraction and amplification. The *B. thuringiensis* sequence *cry*I was detected in all samples and by using both (DH and TSPE-UH) microarray formats. *Y. pestis* signature sequences were retrieved from all the spiked samples. *C. burnetii* was detected in the goat farm samples. All signature sequences were detected in the surface area swabs, while the *icd* and *com* signatures were not detected in the vaginal swab sample. These data are congruent with qPCR results [15].

## Discussion

### Biothreat microarrays

There is an increasing demand for methods which can screen samples for the presence of multiple pathogens. In clinical applications, such multiplex detection methods save valuable time and enhance the chance of identifying mixed infections in patients [8]. When screening samples at high-throughput, for instance for environmental surveys or population studies, multiplexed detection is indispensable for restraining the amount of effort, materials and costs. We developed diagnosic microarrays for sensitive and specific detection of the biothreat pathogens *B. anthracis*, *Y. pestis*, *F. tularensis* and *C. burnetii*. Parallel detection of several signature sequences made it possible to confidently identify these pathogens and assess their virulence potential. False-positive and false-negative measurements are minimized by the inclusion of at least three different signatures for each pathogen. Signatures that return MFI values exceeding predetermined thresholds were considered positive, but a positive signature requires confirmation by complementary pathogen signatures, since cross-reactivity cannot be excluded. Even if diagnostic regions have been carefully selected, the dynamic nature of microbial genomes and the enormous diversity of environmental sequences, warrants the use of redundant, confirmatory signatures [16], [17]. This in-assay confirmation of results also reduces false negatives as unanticipated changes in the selected signatures cannot be ruled out completely. False-negatives are further prevented by the high sensitivity that was attained and by the inclusion of a probe for a signature sequence from *B. thuringiensis*, a near relative of *B. anthracis*. When added to samples before DNA extraction, these highly refractory biological structures serve as internal control for efficient DNA extraction, amplification and microarray detection.

### Signatures for the identification of biothreat pathogens

Detection of the four pathogens was based on the detection of genes and insertion sequences located on the chromosome and on plasmids. Plasmids can be very important for virulence, although plasmid-deficient *B. anthracis* and *Y. pestis* strains exist [14]. Such strains, as well as yet uncharacterized closely related environmental species, share genomic traits that could lead to misidentification. For instance, *B. cereus* strains have been described that carry plasmids highly similar to those of *B. anthracis* [18]. Chromosomal signatures were included for each pathogen as a stable marker, while microarray sensitivity was enhanced by including signatures from multicopy insertion sequences *IS1111* (*C. burnetii*) and *ISFtu2* (*F. tularensis*), or located on multicopy plasmids pXO1 (*B. anthracis*) and pPCP1 (*Y. pestis*).

Besides increasing detection confidence by providing complementary information, multiple signatures for each pathogen also presents valuable information about virulence. The presence of plasmids pXO1 and pXO2 in *B. anthracis* is highly relevant for its virulence as all highly virulent strains possess pXO1 and pXO2, while avirulent vaccine strains do not carry pXO1 or pXO2 [19]. *Y. pestis* contains three plasmids, pPCP1, pMT1 and pCD1, of which the latter also occurs in related *Yersinia* species. Strains devoid of 1 or more plasmids occur regularly, and although the plasmids play a role in pathogenesis, plasmid-cured strains may still be virulent [20]. *F. tularensis* subspecies are known to vary in their virulence and the four recognized *F. tularensis* subspecies could be differentiated based on probes *wbk*, *pdf* and *pdm* (Table S2). Detection of the *ISFtu2* target in one of two *F. philomoragia* strain was consistent with the occurrence of somewhat homologous sequences in some strains of this species, and with its detection by using qPCR [14]. The presence of the *Y. pestis* specific *pla* gene in rat samples was not caused by cross-reactivity of this probe but by the presence of a highly homologous sequence in these rats as was reported previously [14].

### Comparison DH and TSPE-UH assay formats

To be useful for diagnostic and screening purposes, microarrays demand above all sensitivity and specificity. These qualities are largely determined by oligonucleotide probe selection and design, which control for instance probe melting temperature and potential cross-hybridization. In addition, sensitivity and specificity can be affected by the assay chemistry which determines the conditions in which the designed oligonucleotides hybridize to target DNA to enable amplification, labeling and microarray binding. The assay format also has implications for other parameters determining microarray applicability, i.e. speed, flexibility, multiplexing capability, ease-of-use and cost [3]. We investigated the effect of alternative assay chemistries on microarray performance, particularly with regard to specificity and sensitivity. Two assay formats were developed that differ in the way pathogen DNA is labeled and hybridized before detection by using suspension microarrays. The initial PCR amplification of target DNA has a major effect on microarray performance, but since this is an essential step regardless of the assay format, this step was not varied.

### Microarray specificity

Consistent cross-reactivity between signatures of different pathogens could potentially lead to false positive detection. This was observed in a few instances. Probe *ypo* (*Y. pestis*) could cross-react with *B. anthracis* and probe *isf* (*F. tularensis*) with *Y. pestis* when using DH microarrays (Fig. 2). In the TSPE-UH microarrays, probe *pl3* could cross-react with *Y. pestis*. However, these cross-hybridization signals were low and occurred only if the target concentrations were very high. Therefore, only in the presence of a significant amount of one of the targeted pathogens, PCR amplification may lead to an erroneous signal. This is unlikely to occur during normal sample screening. Moreover, due to the absence of complementary pathogen signals, this will not lead to false positive detection of a secondary pathogen, especially if the researcher is aware of the potential occurrence of this cross-reactivity. On the other hand, the cross-reactivity of the *com* primer with multiple targets in the TSPE-UH microarrays was to such an extent that its applicability was disqualified. The microarrays included sufficient complementary signatures for reliable detection of *C. burnetii*. We included the *com* findings in our report to illustrate the differences in specificity that may occur when the oligonucleotide is used as primer (TSPE-UH) or as hybridization probe (DH).

### Scoring positive samples

The signal-to-background ratios obtained from microarray measurements were usually very high (Fig. 1, 2, 3, Table S2), which made recognition of positive signals straightforward. Nevertheless, there was variation between experiments and some DH measurements showed relatively low MFI values for hybridized probes or high background signals. Although the MFI values for DH microarrays were usually higher, the signal-to-background ratios were lower due to the relatively high background signals. The higher and more variable background signals in DH microarrays (Fig. 1, Table S2) may have been due to probe quality, variation in coupling efficiency of the probes to the beads, and variation in hybridization efficiency of target DNA to the probes. The production of absolutely biotin-free probes presents a challenge and traces of biotin may have contributed to the higher background signals in the DH microarrays. In contrast, TSPE-UH microarrays use xTAG coupled beads which are standardized by the manufacturer. These beads had lower and more constant background signals compared to the DH probes. We defined different scoring rules for differentiation between hybridized and non-hybridized probes for both microarray formats. The scoring rules were based on several experiments during our study period, including measurements using novel batches of bead-coupled probes. The signal-to-background ratios were very high for probes hybridizing to their intended targets. This was also true for pathogens at the detection limit (LOD experiments; data not shown) or constituting only a minor fraction of the sample (Fig. 3). Therefore, varying the rules for scoring positive signals does not affect the outcome of experiments measuring LOD (Table 2) or mixed pathogen DNA (Fig. 3). On the other hand, changing these rules did affect the detection of positive signals for some probes (Table S2). Therefore, although these rules were suitable for our set of experiments, it may be necessary to adjust the rules in another set of measurements.

Wilson *et al.* [6] reported a correlation between input DNA and the resulting MFI signal. The MFI signals they reported were considerable lower than in our results, especially for low DNA input. These microarray readouts require a robust protocol for differentiation between hybridized samples and background, and it may be difficult to confidently detect low amounts of DNA. In contrast, we did not observe a relation between DNA target concentration and MFI signals and the signal-to-background ratio was high, also at low DNA input. The only exception is probe *wbk*, which displayed a lower signal at lower DNA input in the DH microarrays (Fig. 3). The high MFIs and absence of a relation with target DNA input is probably due to the production of labeled single stranded DNA in our microarrays which efficiently saturates the bead-coupled probes. As a consequence, the microarray signals cannot be used quantitatively. A benefit is that low DNA input does not impair detection signals and scoring positive samples is much more straightforward.

### Microarray sensitivity

Microarray sensitivities are usually measured by replicate 10-fold dilutions, yet we performed Probit analyses to estimate the lowest target concentration at which there is a 95% chance of producing a signal. Probit repression analysis is often used for estimating the LOD of qPCR assays [21], [22], [23]. It needs to be stressed that around the detection limit, data do not comply with the requirement of normal distribution, which may affect the accuracy of the LOD estimates. Nevertheless, the calculated LODs can be used to compare the efficiencies of different probes and assay chemistries and provide a good estimation of the sensitivity of the method.

Probe hybridization efficiency differed between probes and was highest for *isf*, *caf* and *IS1*, and lowest for *wbk*, *icd* and *ypo*. Although detection by using the TSPE-UH format seemed to be slightly more sensitive, the LODs for the target amplicons were largely comparable between both assay formats (Table 2). This indicates that the assay chemistry did not have a significant effect on microarray sensitivity.

The sensitivities measured for the detection of genomic DNA are the result of probe binding, but also of target copy number. The *B. anthracis* strain we used does not contain high copy numbers of the pXO1 and pXO2 plasmids [14], which explains the similar LODs based on plasmid and chromosomal signatures. For *F. tularensis*, sensitive detection based on the *isf* signature can be explained by its presence in multiple copies per genome combined with efficient probe hybridization (as evidenced by the low LODs from single amplicons). Also, sensitive detection of *Y. pestis* based on *pla* is explained by its multiply copies per genome. Efficient hybridization (low LOD single target amplicons) did not always correlate to sensitive detection of genomic DNA as illustrated by the relatively high LOD of *caf*.

The LODs in Table 2 can be used to calculate a concentration of approximately 3 genomic equivalents (GE) of *F. tularensis* per sample that can be measured with 95% confidence. Calculating the number of GE from the amount of gDNA presents problems for the other pathogens, due to the variable and unknown contribution of plasmid DNA in *B. anthracis* and *Y.pestis*, and of host cells DNA in *C. burnetii*. Nevertheless, by using an estimated DNA content of 5.8 Mbp for *B. anthracis* and 5.0 Mbp for *Y. pestis*, LODs of approximately 5 GE could be calculated for both pathogens. Evidently, more DNA is required for confirmation by other signatures.

The LODs for most signatures in the microarrays were slightly lower than those obtained from multiplex qPCR assays that were developed previously [14], [15]. However, this varied between signatures, and the resulting LOD for *Y. pestis* was similar to qPCR, for *B. anthracis* somewhat higher (factor 2), and for *F. tularensis* considerably higher (factor 10). The LODs for genomic DNA displayed in Table 2 represent the upper limits of the LODs, i.e. the assays may actually be more sensitive. This is due to the method that was used for measuring DNA concentrations, which may overestimate the DNA content in genomic DNA preparations due to the presence of RNA. Previously, it has been reported that the LATE-PCR rules are ineffective for multiplex asymmetric PCR amplification [24]. Yet, our results show that it is possible to develop a 16-plex asymmetric PCR for the production of single-stranded DNA, which allows highly sensitive detection. It is difficult to compare the LODs calculated by using Probit analysis directly to LODs based on assessment of replicate measurements of 10-fold target DNA dilutions, which is the method that is usually applied. Nevertheless, the LODs of the assays described here were lower compared to those of other microarrays for the detection of biothreat pathogens [6], [25] or other micro-organisms [24]. Microarrays for the detection of biothreat agents included a 10-plex assay with detection limits of 10^2^–10^4^ GE [6], and a 13-plex assay with detection limits of 10^3^–10^4^ GE [25]. Increased performance may be due to specific primer design, the use of a special multiplex PCR formula, and the application of asymmetric PCR which improves the detection.

### Mixed pathogen detection

One of the advantages of a multiplexed diagnostic array is its capability to detect more than one pathogen in the same sample. Application of a diagnostic microarray for the detection of respiratory infections has shown that this promotes the detection of secondary infections which otherwise might go undetected [8]. Also for biothreat surveillance it may be beneficial to be able to detect multiple pathogens simultaneously, as deliberate release of mixed pathogens is not unthinkable. Our results show that the presence of mixed pathogens DNA did not inhibit detection, up to the tested ratios of 1∶100 (Fig. 3).

### Environmental testing

The detection of pathogen signature sequences in a few different sample types confirmed that the developed microarrays could be applied for screening purposes.

The samples spiked with *Y. pestis* DNA were representative for a few common matrices that could be encountered when screening veterinary or environmental samples for the presence of multiple pathogens. In addition, it was shown that the microarrays could be used to detect *C. burnetii* DNA in natural samples that were collected on goat farms that were linked to human cases during an ongoing Qfever outbreak in the Netherlands [15]. The detection of only the multicopy (IS1111) and one other (*ser*) signature in the vaginal swab sample can be explained by low amounts of *C. burnetii* DNA. More sensitive detection of *IS1111* is explained by its presence in multiple copies, while the detection of ser and not the other single-copy sequences may be due to the somewhat higher sensitivity of the ser probe (Table 2).

### Conclusions

The microarrays that were developed enable confident multiplex detection of the biothreat pathogens *B. anthracis*, *Y. pestis*, *F. tularensis* and *C. burnetii*. Both microarray formats offer high specificity and a sensitivity that is only slightly lower than that of qPCR detection. The major benefit of using microarrays is their multiplexing capability. The use of asymmetric multiplex PCR in combination with DH microarrays, and of TSPE-UH microarrays produce high signal-to-background ratios, also for low amounts of input DNA. This makes the recognition of hybridized probes straightforward. Multiple pathogens can be detected simultaneously, also if present in different ratios.

Differences between the applicability of both microarray formats are related to practical issues and to inherent benefits and disadvantages. DH microarrays must be synthesized in-house, which was responsible for higher and more variable background signals compared to the TSPE-UH microarrays. This increases the variation between experiments. Furthermore, The DH protocol requires an asymmetric PCR step which was considerably slower than conventional PCR, when following the protocol in this report. However, this protocol can be optimized significantly (as will be reported elsewhere). The TSPE-UH does not require asymmetric PCR but includes an additional 2 hours of primer extension and labeling. Overall, the TSPE format takes more time. On the other hand, an advantage of the TSPE-UH microarray format is its flexibility as it is more convenient to modify probes (which do not need to be coupled to the beads).

## Materials and Methods

### Bacterial isolates and genomic DNA preparation

The detection limits and specificities of the assays were evaluated using genomic materials from the bacterial strains and other sources displayed in Table S2. The pathogen panel included (besides a variety of *Eukaryal* organisms): 8 *B. anthracis* strains and 14 near relatives (8 *B. cereus*, 5 *B. thuringiensis* and 1 *B. mycoides*), 21 *F. tularensis* strains (8 subspecies *holarctica*, 4 *tularensis* and 1 *novicida*) and 2 of the closest related species *F. philomiragia*, 13 *Y. pestis* (including *Antiqua*, *Mediaevalis* and *Orientalis* biovars) and 3 strains from the closest relative *Y. pseudotuberculosis* and 3 strains from *Y. enterocolitica*. From *C. burnetii* we had one reference strain available. From most of the *B. anthracis*, *F. tularensis* and *Y. pestis* strains and from the *C. burnetii* strain we had genomic DNA (lysates) available to verify specificity of our assays. Several strains were available as live cultures in our laboratory and these were used as resource for the production of larger quantities of genomic DNA. *B. anthracis* and *Y. pestis* strains were acquired from the NCTC (National Culture Type Collection, UK) and the Pasteur Institute (France). The *Francisella holarctica* strain was isolated from a patient at Slotervaart Hospital, Amsterdam, and kindly given to us for research purposes. Patient consent was obtained at the time of sampling. Cultivation of these strains was carried out in a BSL3 glove-box as described previously [14]. Cultures from non-target bacteria that were used in the specificity panel were obtained from the culture collection at the RIVM. These cultures were cultivated under BSL2 conditions and lysates of these cultures were used for specificity testing.

Other genomic materials were lysates from bacterial cultures provided by other researchers as mentioned in the acknowledgements. Genomic material from *C. burnetii* strain Nine-Mile was obtained from Virion (Institut Virion\Serion, Serion Immundiagnostica, Würzburg, Germany). DNA extraction and purification was carried out by using NucliSens Magnetic Extraction Reagents (bioMérieux, Boxtel, the Netherlands) as described previously [14]. DNA concentrations were measured using the NanoDrop 1000 spectrophotometer (Thermo Fisher Scientific, Wilmington, USA). DNA samples were stored at 4°C for use within 1 week and at −20°C for longer storage.

### Signature sequences

Criteria for the selection of signature sequences for pathogens were as described previously [14]. The signatures for *B. anthracis*, *F. tularensis* and *Y. pestis* described in this reference were selected for the design of primers and probes. Additional signature sequences included the following. Four signature sequences, including a multicopy insertion sequence, were selected for the detection of *C. burnetii*. Signature sequence *serS* was developed to supplement *comI*, which appeared to cause cross-reactivity in the TSPE-UH assays. To improve the coverage of *Y. pestis* strains, the chromosomal signature sequence *yihN* sequence [26] was added to the *YPO393* sequence that had been developed originally, as the latter was found to be absent in a few *Y. pestis* strains from a Nairobi cluster [14]. For *C. burnetii*, two chromosomal sequences were used since strain diversity could be considered only to a limited degree due to the scarcity of sequenced genomes and strains available for validation.

To improve differentiation of highly virulent *Francisella tularensis* strains, a signature sequences was identified, that is only present in the virulent subspecies *tularensis*, *holarctica* and *mediasiatica*. This sequence was identified by using the Insignia genome comparison tool (http://insignia.cbcb.umd.edu). The genome from *F. tularensis* tularensis strain Schu S4 was selected as the reference, and genomes of all 5 tularensis, 6 *holarctica* and 1 *mediasiatica* subspecies as additional target genomes. The genomes of the avirulent subspecies *novicida* and related species *F. philomiragia* were thus excluded. Unique and conserved signature sequences identified by Insignia were inspected and a BLAST search (http://ncbi.nlm.nih.gov/BLST/) was performed to confirm specificity for the selected strains. A portion of the gene *wbtK* was thus selected for probe design, as it is an annotated gene of sufficient length. Differentiation of *F. tularensis* subspecies can be based on the hybridization pattern of the wbtK and pdpD signatures. The *pdpD* gene is present in subspecies *tularensis*, *novicida* and *mediasiatica*, and has an insertion of approximately 150 bp in the latter two [27]. Probe *pdf* hybridizes to the pdpD gene, while *pdm* specifically targets the insertion sequence. No signal from both *pdpD* probes points to subspecies *holarctica*, since this subspecies does not carry this gene [27]. Subspecies tularensis is present when probe *pdf* is detected while *pdm* is not, and subspecies *mediasiatica* is present when probe *pdm* is detected. Subspecies *novicida* also yields a signal for *pdm* (and for *pdf*), but, as mentioned above, lacks the *wbk* gene. Probe hybridization was tested by using DNA from various pathogen strains. However, the *F. tularensis mediasiatica* hybridization pattern was confirmed by *in silico* validation (strain FSC 147, accession CP000915) only, since we did not have genomic materials available from this subspecies.

### Probe and primer design

From each signature sequence, a comprehensive alignment was constructed from all available NCBI/EMBL entries by using the software package Kodon (Applied Maths, Ghent, Belgium). Oligonucleotides for multiplex (a)symmetric PCR and for microarray probes were designed using the software package Visual Oligonucleotide Modeling Platform version 6 (DNA software Inc. Ann Arbor, USA). For each signature sequence, specific probes were designed first. TSPE primers were constructed by extending these probe sequences with unique TAG sequences. Subsequently, a corresponding primer pair was designed for multiplex PCR amplification. The primer concentration for the primer annealing to the same strand as the probe was set at 10 nM and for the other primer at 200 nM. For validation of the amplification and probe hybridization of each separate signature sequence in the microarray, we produced amplicons sizing 400–800 bp. These amplicons extended at least 50 bp beyond both ends of the region amplified by the multiplex PCR described above. Primer sequences are displayed in Table S1. After amplification, PCR products were purified and the number of amplicon copies was calculated from their sizes and concentrations. Oligonucleotides candidates that were calculated by the design software were first checked against the consensus alignment to exclude designs not covering all sequence variants, and were then evaluated using the simulation module of Visual OMP. All oligonucleotides were validated in silico by using BLAST searches in general and microbial genomes databases (NCBI/EMBL).

### PCR amplification

Oligonucleotides for PCR were synthesized by Biolegio (Biolegio, Nijmegen, the Netherlands). Conventional PCR was used to produce amplicons from signature sequences. Amplification was carried out using the HotStarTaq Master Mix Kit (Qiagen, Westburg, the Netherlands) and 400 nM primers in a total reaction volume of 50 µl. Thermocycling conditions were as follows: 95°C for 15 min, 40 cycles at 95°C for 30 sec, 55°C for 30 sec and 72°C for 30 sec, followed by a final step at 72°C for 7 min. Thermocycling reactions were carried out in a iQ5 thermal cycler (Bio-Rad).

Multiplex PCR reactions were carried out in a final volume of 20 µl containing Qiagen Multiplex PCR mix (Qiagen, Westburg, the Netherlands) with 2 µl DNA template added to the reaction. Initially, experiments were carried out using 200 nM primer concentrations. After changing the protocol into asymmetric PCR, the biotin-labeled excess primer was used at a final concentration of 200 nM, while the limiting primer was used at 20 nM final concentration. Experiments had shown that a concentration of 20 nM instead of 10 nM for the limiting primers yielded good results while fewer PCR cycles were required. The thermal cycling conditions were as follows: First enzyme activation at 95°C for 15 min, followed by 50 cycles of 95°C for 30 sec, 57°C for 90 sec and 72°C for 90 sec, and a final incubation at 72°C for 10 min. Each experiment included a negative (no template) control. Amplification was carried out on an iQ5 (Bio-Rad) instrument. Verification of PCR products were carried out on the Agilent 2100 Bioanalyzer instrument using the DNA 1000 kit (Agilent Technologies, Eindhoven, the Netherlands).

### Direct Hybridization

Amino-modified, 5′ C-12 coupled and biotin-free probes were obtained from Metabion (Metabion Gmbh, Martinsried, Germany). Magnetic beads (MagPlex Microspheres) were obtained from Luminex corporation (Austin, Texas, USA). Probes were coupled to the beads according to the manufacturers recommendations. The beads were counted by using microscope counting chambers, diluted to equal concentrations and stored in the dark at 4°C. Hybridization and measurements were carried out following the manufacturers recommendations. Briefly, beadmixes were composed containing beads of each type at a concentration of 1000 beads/µl. The beads were diluted 10 times in 1.5× TMAC hybridization solution. PCR amplified and biotinylated DNA was incubated with the bead mix at 55°C for 15 min, followed by incubation of the beads with a reporter mix containing streptavidin-R-phycoerythrin and incubation at 55°C for 5 min. Beads were collected by using a magnetic separator during these steps. Analysis was carried out on the Luminex 100 instrument according to the system manual.

Each measurement included a no template PCR control, and a control for beadsignal performance which constituted of a mixture of biotinylated reverse-complement probes.

### Target Specific Primer Extension and Universal Hybridization (TSPE-UH)

TSPE for labeling and TAG incorporation was carried out as follows. The same biotinylated asymmetric PCR products as described in the DH protocol above were used as templates for target specific primer extension (TSPE reaction). Although the TSPE-UH protocol works equally well with standard PCR amplification and labeling occurs in the subsequent TSPE reaction, we used the same PCR products for both assay chemistries since this allowed direct comparisons and limited the amount of amplification reactions. Primer extension was carried out in a 20 µl final reaction containing 5 µl of the multiplex (asymmetric) PCR products, 0,75 U Platinum GenoType *Tsp* DNA polymerase (Invitrogen), 1× TSPE buffer (20 mM Tris-HCl; pH 8,4; 50 mM KCl), 1,25 mM MgCl_2_, 5 µM dATP, dTTP, dGTP, 5 µM biotin-dCTP, and 2 nM of each TAG-TSPE primer as described above. Thermocycling conditions were as follows: 96°C for 2 min, 30 cycles at 94°C for 30 sec, 55°C for 1 min and 74°C for 2 min. Thermocycling reactions were carried out in a iQ5 thermal cycler (Bio-Rad). Beads coated with anti-TAG probes (MicroPlex™ –xTAG™) were obtained from Luminex Corporation. TAG-TSPE primers were ordered from Biolegio (Biolegio, Nijmegen, the Netherlands). Hybridization and measurements were carried out guided by the manufacturers recommendations. Briefly, a beadmix was composed by combining MicroPlex beads to a concentration of 2500 beads of each set per reaction. 5 µl TSPE reaction products was added to each reaction and the total volume was brought to 50 µl. The samples were denatured at 96°C for 90 sec followed by hybridization at 37°C for 30 min and washing. Beads were incubated with 2 µg/ml streptavidin-R-phycoerythrin in hybridization buffer at room temperature for 15 min and analyzed on the Luminex 100 instrument according to the system manual. Each measurement included a no template PCR control.

### Microarray performance: limit of detection (LOD) and specificity

Calculations of the LOD were based on dilutions of genomic DNA (gDNA) from representative pathogen strains, as well as on dilutions of purified PCR amplicons. These PCR amplicons included >50 bp upstream and downstream sequences from the multiplex PCR amplification sites and were used to compose template mixes of desired composition and quantities, while maintaining secondary structures in the primer binding regions. Representative pathogen strains used were *B. anthracis* strain Vollum, *F. tularensis* strain *tularensis* ATCC 6223, *Y.pestis* strain Harbin and *C. burnetii* strain Nine-Mile (Table S2). DNA was purified from lysates of these strains. The concentrations of purified gDNA and of purified PCR amplicons were measured by using the NanoDrop 1000 spectrophotometer. Since *C. burnetii* was cultivated in cell lines, its lysates contain an unknown but likely significant portion of host cell DNA. Therefore, it was not possible to measure the gDNA concentration reliably and we only calculated the LOD from PCR amplicons. Serial dilutions of DNA were used to calculate LODs from the proportion of positive signals at each dilution. Five replicates of nine serial dilutions were measured in the microarrays. The measurements included at least one dilution with all replicates positive and one with all replicates negative. A probit analysis was performed using SPSS Statistics 18.0.0 to calculate the DNA concentration that could be measured with 95% probability. Specificity of the developed microarrays was assessed by measuring DNA from the specificity panel containing pathogen strains, related organisms and various other Bacteria and Eukarya (Table S2).

### Microarray performance: mixed pathogens and environmental samples

Microarray detection of multiple pathogens simultaneously was investigated by using mixed gDNA from the representative *B. anthracis*, *F. tularensis* and *Y. pestis* strains mentioned above. Mixed DNA was used from three combinations of two pathogens (*B. anthracis* and *F. tularensis*, *B. anthracis* and *Y. pestis* and *F. tularensis* and *Y. pestis*). Each combination was mixed in the ratios 200∶2, 20∶2, 2∶2, 2∶20, 2∶200 (pg per reaction). In addition, a mixture was made of all four pathogens in a ration of 1∶ 1∶1∶1 (pg per reaction).

The possibility to detect the pathogens in environmental samples was investigated by performing microarray measurements on different representative sample types spiked with pathogen DNA and by measuring samples from a goat farm which was known to be contaminated with *C. burnetii* from studies using qPCR. In the samples used for spiking, 10^3^ pg DNA from *Y. pestis* strain Harbin was added to blood (EDTA preserved goat blood), faeces (goat faeces that had been mixed with PBS and incubated overnight at 4°C), surface water extract (filter extract concentrated from approximately 200 L surface water, as described in [28] or coffee creamer powder. As an internal control, 50 µl of a B. *thuringiensis* spore suspension (1,2×10^5^ spores) was added to each sample. DNA was extracted by using the NucliSens Magnetic Extraction Reagents (bioMérieux) according to the manufacturers instructions. The samples from the farms contaminated with *C. burnetii* were collected at two different goat farms in October 2009. Surface area swabs collected in the stables and vaginal swabs of animals were collected using sterile cotton swabs (VWR International, the Netherlands). Swabs were added to 10 ml of NucliSens lysisbuffer from the NucliSens Magnetic Extraction Reagents (bioMérieux) and vortexed. As an internal control, 50 µl of a *B. thuringiensis* spore suspension (1,2×10^5^ spores) was added to each sample and the samples were incubated for one hour. Subsequent DNA extraction was carried out according to the manufacturers instructions.

## Supporting Information

Table S1 **Primers used for the production of amplicons from signature sequences.**
(PDF)

Table S2 **Panel of organisms that was used for validation of the DH and TSPE-UH microarrays.** Values displayed are MFI values. Highlighted are values that exceed the threshold as described in the text. The *com* probe is not included as its use for specific detection was invalidated.
(PDF)
