# Supplementary material for: Development and Comparison of Two Assay Formats for Parallel Detection of Four Biothreat Pathogens by Using Suspension Microarrays
Source: PLoS One. 2012 Feb 15;7(2):e31958. doi: 10.1371/journal.pone.0031958 (PMC3280232; doi:10.1371/journal.pone.0031958)
Supplement: Table S1 — Primers used for the production of amplicons from signature sequences. (PDF) [file pone.0031958.s001.pdf]

Supplemental table S1 - Primers used for the production of amplicons from signature sequences

Target	*Oligo name*	*Sequence 5'-3'*
pl3	pl3trg_f	ATTTTCAAAAAAACCTGTACTGGAAGAG
	pl3trg_r	GTATCTTGCAATGTAATCATAAGCTCATGT
cya	cyatrg_f	CTGAAAGGAGAAAAAGCACTTAAAGCTTC
	cyatrg_r	TAGTTGAATCCGGTTTCCTCTCAATTC
capB	cabtrg_f	CGCGAATGATATATTGGTTTACTGACG
	cabtrg_r	CGGTCAGGGCGGCAA
ypo0393	ypotrg_f	AAACACCTTTCCCTATAGATCGTACTG
	ypotrg_r	AAATCTTCTTCTCAAAACAAAGAAAAACAT
yinH	yintrg_f	GCGCTTACGGGATCATTAATCAGT
	yintrg_r	CTGCACGCATTCTTTTTGTTAAAATAGTGG
Pla1	platrg_f	ATGAAAATCAATCTGAGTGGACAGATCAC
	platrg_r	CAGAAGCGATATTGCAGAC
caf1	caftrg_f	TGCATTATTTGGAACTATTGCAACTGCTA
	caftrg_r	CCGCCTTTGGAACCAATTGAGC
fopA	foatrg_f	GATGAGATTAAAAAGTATTGTTATAGCTAC
	foatrg_r	CTGCAGCATATGGAGTAAACATAGTAT
wbk	wbktrg_f	GCAACTCCGCGTAAAGTGTCTGATAA
	wbktrg_r	GTCAATGTTTAGAGTCTTTGGTTACTCAAG
ISFtu2	isftrg_f	ATCATTCTTAAAATCACAAAAAGGTATACA
	isftrg_r	ATACCTTGAATATGCTGCCTGATTTCATT
pdpD	pdptrg_f	CTGATGACTTAATTGGTTTTTCAGATGCTA
	pdptrg_r	GTTTGTGAGTAGTTAATTAAAGTATCTTGAAC
com1	comtrg_f	CCCTGCAATTGGAACGAAG
	comtrg_r	GTTCTGATAATTGGCCGTCGACA
ser	sertrg_f	CCACTTCGTTGGCCTCCC
	sertrg_r	ACACGAAGCTGCCACCT
IS1111	is1trg_f	GGGCTGCGTGGTGATG
	is1trg_r	GCGCCATGAATCAATAACGTCCTTA
icd	icdtrg_f	CGGAGTTAACCGGAGTATCCA
	icdtrg_r	CCGTGAATTTCATGATGTTACCTTT
Cry1	crytrg_f	CACCATTATCACAAAGATATCGGGTAAGA
	crytrg_r	TCAGTTCGTCTGTATTGTTCTCGATC
