# Supplementary material for: Development and Comparison of Two Assay Formats for Parallel Detection of Four Biothreat Pathogens by Using Suspension Microarrays
Source: PLoS One. 2012 Feb 15;7(2):e31958. doi: 10.1371/journal.pone.0031958 (PMC3280232; doi:10.1371/journal.pone.0031958)
Supplement: Table S2 — Panel of organisms that was used for validation of the DH and TSPE-UH microarrays. Values displayed are MFI values. Highlighted are values that exceed the threshold as described in the text. The com probe is not included as its use for specific detection was invalidated. (PDF) [file pone.0031958.s002.pdf]

Table S2. Specificity panel DH microarray

highlight = value above threshold

			B. anthracis probes			*F. tularensis* probes					*Y. pestis* probes				*C. burnetii* probes			IC
**Species**	**strain ID**	**origin**	**pl3**	**cya**	**cab**	**foa**	**isf**	**wbk**	**pdf**	**pdm**	**ypo**	**yin**	**pla**	**caf**	**ser**	**is1**	**icd**	**cry**
Bacillus anthracis	NCTC 109	Shaving brush, London, 1920	14874	939	1873	474	778	417	935	630	771	486	505	666	532	757	621	647
	NCTC 8234	Weybridge, 1951 (Sterne)	7660	9424	1033	862	1211	575	1482	717	1716	654	656	1085	893	1281	936	1151
	NCTC 7753	Bradford, 1948	14441	2171	22746	423	858	398	922	458	961	550	452	715	573	820	653	757
	NCTC 7752	Bradford, 1948	15574	1974	22066	416	743	358	787	372	863	441	353	727	502	725	571	616
	NCTC 5444	St Marys Hospital, London, 1938	14630	2128	21265	456	869	404	929	360	1008	593	405	713	544	790	618	753
	NCTC 2620	Chinese hide, London, 1928	7483	11917	12926	485	845	379	945	416	931	535	428	784	581	807	671	754
	NCTC 1328	London, 1922	11803	16200	15371	477	863	413	894	469	1940	558	445	778	543	752	670	739
	NCTC 10340	Cow, Edinburgh, 1963 (Vollum)	4386	5393	5076	868	1322	550	1421	781	1388	731	810	1109	796	1335	1036	1316
Francisella tularensis subsp. tularensis (A	ATCC 6223	Human lymph node, Utah USA, 1920	561	615	524	24025	10127	20643	6155	346	596	420	447	546	1054	643	521	625
	HFSC4	Dugway, Utah, USA	1298	1158	976	28026	13874	22025	9967	853	1982	1623	1936	1446	3400	1462	1301	1493
	HFSC15 (SR/1)		1192	1088	884	28570	13726	23537	10531	601	2074	1431	1863	1233	3474	1424	1398	1206
	SEV23	Ixodes tick, Australia, 1990	996	904	1098	28382	14169	23089	10405	808	1976	1304	1695	1129	3128	1968	1325	1051
Francisella tularensis subsp. holarctica (B	BD07-537	Clinical isolate, Netherlands, 2007	995	1305	1104	6786	4241	6453	1716	713	1290	726	921	1214	1026	1281	1011	1035
	LVS	Live vaccine strain, Russia	1442	1623	1471	28947	12802	18828	1730	1057	2326	1787	1819	1467	3102	1385	1169	1999
	T8	Tick, Japan, 1957	944	1001	1019	28223	13549	11600	1133	683	1842	1098	1369	1023	1733	1192	889	1179
	T21 (FSC089)	Human blood, Norway, 1989	1225	1468	1076	28224	13911	18321	1406	1099	2177	1147	1908	1300	2782	1317	1381	1474
	T20	Clinical isolate, France, 1994	1038	1344	1084	28228	13477	23259	1520	832	1480	1303	1617	1254	2584	1382	2203	1266
	T4	Vaccin strain SBL vaccin	1446	1135	1160	28413	12881	20720	1240	876	2192	1571	1737	1629	3174	1442	1684	1818
	T23	Clinical isolate, Sweden, 1998	1420	1090	1053	28818	13658	22490	1666	1012	2370	1475	2042	1459	2908	2093	1445	1427
	T11	Clinical isolate, Sweden, 1984	1379	1351	1280	27850	13331	20409	2888	720	2293	2347	1484	1468	3271	1494	1815	1517
Francisella tularensis subsp. novicida	ATCC 15482	Water, USA, 1951	1020	1153	853	15785	9874	490	4713	15350	1031	713	739	926	855	1214	970	1000
Yersinia pestis	Kenya 164	Biovar antiqua, Kenya, <1952	1404	1393	1230	995	1900	696	1804	958	6274	5853	6778	5903	986	1663	1302	1564
	Harbin	Biovar mediaevalis, China, <1948	1718	1365	1460	1472	3940	1256	1833	1088	24860	24038	26643	24626	1546	1520	1257	1861
	KIM3	Biovar mediaevalis, Kurdistan, 1968	795	685	697	1519	3044	1531	791	585	24556	22975	965	24584	1274	856	1123	868
	Madagascar 34-94	Biovar orientalis, Madagascar	1619	1466	1398	1309	3834	1306	1700	1050	25211	23762	27301	25401	1497	1587	1370	1716
	Madagascar 73-93	Biovar orientalis, Madagascar	1048	890	1041	1933	3133	1329	950	819	23772	22413	26035	24505	1247	1017	1253	1129
	Nha Trang 63-115	Biovar orientalis, Vietnam	665	675	608	450	824	435	897	444	19204	15652	23654	743	546	784	580	677
	Hamburg 19	Biovar orientalis, Hamburg	790	721	670	445	1671	427	862	363	19612	15687	400	18617	524	788	677	763
	EV 76	Biovar orientalis, Madagascar	773	705	707	489	1248	406	901	456	18632	14806	23578	16504	551	774	652	783
	Peru 184	Peru	718	666	630	424	1511	394	781	426	19188	16549	23941	18319	523	770	567	678
	NCTC 5923	Java, 1939	912	983	850	1495	3084	1447	939	777	24780	23186	26704	24991	1593	1174	1478	1063
	NCTC 10029	Human bubonic plague, Nairobi, 1958	1799	1552	1575	1592	4118	1378	1939	1201	1638	23731	27314	25252	1722	1581	1784	1465
	NCTC 10030	Human fatal bubonic plague, Nairobi, 1958	1738	1506	1551	1674	3817	1371	2008	1187	1702	23689	27109	25132	1798	1769	1723	1456
	NCTC 10330	Nairobi, 1963	1815	1486	1565	1695	4191	1438	1976	1231	1732	23770	27274	24958	1735	1695	1806	1393
Coxiella burneti	Nine Mile	Dermacentor andersoni (tick), Montana, 1937	1029	1001	802	840	1100	826	931	595	1027	878	725	909	5792	7851	9362	915
	Priscilla	Goat cotyledon, USA, 1980	482	732	377	1442	465	634	470	290	474	429	483	436	23060	24254	23821	653
	48	*Haemaphysalis punctata* (tick), Slovakia, 1967	581	587	393	1170	464	669	460	290	621	470	522	428	22171	23022	24256	511
	S	Hepatitis, chronic Q-fever, USA, 1981	642	535	412	676	514	685	492	319	626	509	531	462	20538	23280	24204	557
	CbuG_Q212	Human hart valve, Nova Scotia, 1981	669	548	424	901	545	787	552	336	662	570	643	487	20491	22439	19719	601
	Henzerling	Human vaccine strain, Italy, 1945	751	693	493	1416	598	884	582	379	736	566	665	508	20670	22526	21158	628
Bacillus atrophaeus	ATCC 9372		857	782	826	551	834	488	1052	572	864	541	534	766	549	797	704	821
Bacillus cereus	ATCC 11778	NCIB Aberdeen, 1962	1005	1033	771	979	1139	608	1422	742	1188	759	662	1119	697	1087	1025	1063
	BD03-229	Clinical isolate, Netherlands, 2003	783	760	685	461	859	396	993	430	768	532	423	767	570	839	624	738
	BD05-273	Clinical isolate, Netherlands, 2005	799	767	741	449	827	355	917	430	742	532	430	686	546	811	636	734
	WSBC 10530	Vomit from cooked rice, USA or UK, 1972	780	723	593	476	848	368	945	435	771	560	431	774	555	812	695	14716
	WSBC 10536	Indian rice dish, Germany/Pasau, 2001	710	744	611	418	760	327	853	370	682	481	389	685	502	746	562	14772
	WSBC 10583	Cooked rice, 1998	748	764	664	435	848	357	939	396	738	503	414	714	556	798	630	16461
	ATCC 10876	Contaminated bottle	742	736	546	420	747	333	891	360	705	473	371	660	521	775	566	652
	ATCC 7064	Blood	794	734	659	453	794	357	978	426	711	497	404	753	517	861	670	5134
	ATCC 14579		744	726	609	450	857	346	897	410	708	534	434	720	564	761	616	775
Bacillus coagulans		Purchased at Raven Labs, USA	856	693	712	545	921	460	872	470	753	576	459	811	593	851	676	14737
Bacillus licheniformis	ATCC 9945	Flour, USA	1440	1468	1255	781	1711	508	1990	758	1474	853	669	1271	1116	1601	1187	1301
Bacillus megaterium	ATCC 8245		790	856	684	445	900	461	968	474	782	547	441	798	632	835	660	14734
	ATCC 14581	Edinburgh, 1963	1440	1379	1335	730	1605	474	1761	625	1374	662	689	1305	818	1500	1075	1277
Bacillus mycoides	ATCC 6462		768	773	656	500	879	388	1031	450	813	551	473	810	587	864	682	13419
Bacillus pumilus	ATCC 27142		787	711	679	456	762	458	998	377	781	446	494	741	521	864	656	671
Bacillus subtilis	ATCC 6633		947	868	645	531	967	411	1191	418	891	457	405	805	602	905	715	860
Bacillus thuringiensis	ATCC 29730	var. galleriae Heimpel	1401	1293	930	779	1351	629	1740	711	1308	799	806	1272	852	1343	1049	2636
	ATCC 10792	var. berliner, Mediterranean flour moth	606	578	528	436	634	417	804	430	586	418	365	626	440	606	533	14496
	BD07-271	var. kurstaki	606	578	528	436	634	417	804	430	586	418	365	626	440	606	533	14496
	BD07-272	var. aizawai	606	578	528	436	634	417	804	430	586	418	365	626	440	606	533	14496
	BD07-273	var. galleriae	606	578	528	436	634	417	804	430	586	418	365	626	440	606	533	14496
Burkholderia mallei	NCTC 10229	Bird, Budapest	959	840	870	545	1039	431	1180	455	946	527	495	947	579	978	774	810
	NCTC 10230		959	840	870	545	1039	431	1180	455	946	527	495	947	579	978	774	810
Burkholderia pseudomallei	NCTC 10274	Blood of fatal human case, Kuala Lumpur, 1961	920	802	742	518	971	476	1153	506	904	542	457	748	648	1004	827	866
	NCTC 10276	Human, Liverpool, 1962	920	802	742	518	971	476	1153	506	904	542	457	748	648	1004	827	866
Clostridium bifermentans	PW14		1135	1104	1089	822	1035	713	1225	839	1106	971	726	1055	944	1033	937	1130
Enterobacter cloacae	NCTC 13168		871	761	622	442	889	365	919	430	680	561	371	677	427	717	552	673
Escherichia coli	ATCC 25922	Clinical isolate, 1946	825	682	631	448	841	292	879	483	681	417	324	725	525	836	615	696
Francisella philomiragia	CCUG 13404	Clinical isolate, Switzerland, 1979	923	1073	957	692	6947	512	1473	574	1195	710	622	1118	716	1130	930	1083
	ATCC 25015	Moribund muskrat, Utah USA	1121	1036	985	620	1238	456	1393	599	1112	560	568	1089	707	1218	883	1064
Legionella anisa	ATCC 35292	Tap water, Los Angeles, 1981	1098	1023	964	590	1091	461	1332	579	1059	650	511	1060	583	1029	932	1004
Legionella pneumophila serotype 1	ATCC 33152	Human lung, 1977	1094	1144	901	564	1155	427	1325	465	949	505	541	947	647	1075	761	973
Pantoea agglomerans	BD94-368	Clinical isolate, 1994	781	689	639	419	802	375	798	410	678	500	376	713	515	750	604	735
Pseudomonas aeruginosa	ATCC 15442	Animal room water bottle	754	688	642	417	806	378	883	369	709	489	386	727	555	798	642	732
	ATCC 27853	Blood culture, 1969	749	654	602	2892	848	992	735	414	1618	808	837	572	1010	953	654	809
Salmonella enterica subsp. Enterica	ATCC 14028	Serovar Typhimurium. Bovine septicaemic liver, 198	746	716	598	455	744	420	966	425	726	426	385	552	429	769	673	628
	ATCC 13076	Serovar Enteritidis	835	727	671	471	795	351	959	385	745	444	427	652	442	775	693	644
Yersinia enterocolitica	BD94-26	Clinical isolate, 1994	838	938	846	497	1000	432	1184	549	943	556	445	829	583	920	800	798
	BD93-563	Clinical isolate, 1993	838	938	846	497	1000	432	1184	549	943	556	445	829	583	920	800	798
	ATCC 9610	Human tissue, France, 1932	838	938	846	497	1000	432	1184	549	943	556	445	829	583	920	800	798
Yersinia frederiksenii	BD89-1545	Clinical isolate, 1998	810	684	686	463	906	411	938	439	787	573	445	774	543	852	626	736
Yersinia pseudotuberculosis	BD90-58	Clinical isolate, 1990	860	855	747	488	869	461	982	498	794	491	482	708	494	771	635	773
	BD90-89	Clinical isolate, 1990	860	855	747	488	869	461	982	498	794	491	482	708	494	771	635	773
	BD90-101	Clinical isolate, 1990	860	855	747	488	869	461	982	498	794	491	482	708	494	771	635	773
Bos taurus	469	Tissue experimental animal, Netherlands, 2009	1658	1624	1470	1033	1720	721	2069	1184	1797	1105	900	1774	1234	1783	1658	1522
Cerastoderma edule (common cockle foot)		Tissue, Netherlands, 2010	638	658	591	435	724	344	817	370	618	471	372	657	509	689	571	630
Chrysops relictus	I	Tissue, Netherlands, 2009	1511	1409	1310	783	1785	492	1857	738	1582	840	662	1515	931	1549	1062	1414
Ensis directus (razor clams foot)		Tissue, Netherlands, 2010	746	727	748	492	839	384	941	448	766	528	453	684	584	807	667	697
Felis catus	35	Blood, Netherlands, 2010	1306	1142	904	736	1577	494	1718	595	1248	700	553	1168	832	1397	1165	1038
Homo sapiens	Volunteer 8	Blood, Netherlands, 2009	1521	1463	1165	820	1767	766	1704	940	1300	822	863	1252	1059	1549	1148	1304
	Volunteer 10	Blood, Netherlands, 2009	1555	1321	1028	957	1468	844	1715	976	1758	1000	1006	1606	1249	1586	1088	1342
Klebsiella pneumoniae	BD05-258	Clinical isolate, 2005	718	718	647	464	839	441	872	382	770	483	418	697	562	782	672	753
Mus musculus		Tissue, Netherlands, 2009	1479	1661	1402	1162	1699	838	1757	1190	1360	1081	1014	1498	1223	1557	1214	1583
Mytilus edulis (mussel foot)		Tissue, Netherlands, 2010	728	677	633	438	772	373	862	401	745	493	418	696	538	751	647	755
Ovis aries	Twello 67	Tissue slaughterhouse, Netherlands, 2009	1630	1434	1230	887	2065	798	1578	1072	1508	955	813	1582	1105	1638	1463	1400
Rattus norvegicus	8604	Tissue, Netherlands, 2009	894	904	789	524	1099	395	1228	482	906	537	518	790	572	973	689	738
	8605	Tissue, Netherlands, 2009	894	904	789	524	1099	395	1228	482	906	537	518	790	572	973	689	738
Rattus rattus	8403	Tissue, Netherlands, 2009	915	781	751	470	910	459	1103	447	864	504	8466	836	598	827	736	842
	8404	Tissue, Netherlands, 2009	915	781	751	470	910	459	1103	447	864	504	8466	836	598	827	736	842

Table S2. Specificity panel TSPE-UH microarray

highlight = value above threshold

			B. anthracis probes			*F. tularensis* probes					*Y. pestis* probes				*C. burnetii* probes			IC
**Species**	**strain ID**	**origin**	**pl3**	**cya**	**cab**	**foa**	**isf**	**wbk**	**pdf**	**pdm**	**ypo**	**yin**	**pla**	**caf**	**ser**	**is1**	**icd**	**cry**
Bacillus anthracis	NCTC 109	Shaving brush, London, 1920	10990	309	296	250	253	261	295	288	295	378	293	265	350	283	266	365
	NCTC 8234	Weybridge, 1951 (Sterne)	9625	2712	296	302	386	383	400	385	238	373	276	339	423	326	266	388
	NCTC 7753	Bradford, 1948	11242	303	4683	302	236	249	237	265	299	286	217	305	259	224	202	280
	NCTC 7752	Bradford, 1948	12154	252	5919	299	287	311	267	316	267	348	244	245	246	238	258	287
	NCTC 5444	St Marys Hospital, London, 1938	11948	342	5720	287	289	343	274	313	237	320	278	304	239	277	267	251
	NCTC 2620	Chinese hide, London, 1928	12208	3729	5538	271	281	298	223	297	244	271	328	295	259	266	282	379
	NCTC 1328	London, 1922	13537	3625	9113	284	252	348	280	302	240	347	291	290	296	241	225	228
	NCTC 10340	Cow, Edinburgh, 1963 (Vollum)	11048	3880	4177	327	291	406	204	395	224	296	322	357	287	288	304	435
Francisella tularensis subsp. tularensis (A)	ATCC 6223	Human lymph node, Utah USA, 1920	208	321	305	9879	3919	7655	11145	373	291	315	319	270	280	306	252	186
	HFSC4	Dugway, Utah, USA	244	299	285	10763	5297	7005	10946	311	268	332	251	354	274	272	240	308
	HFSC15 (SR/1)		254	242	268	10103	3369	5993	11047	220	263	307	254	337	249	295	247	220
	SEV23	Ixodes tick, Australia, 1990	240	234	240	10376	4239	4590	10469	246	251	344	275	285	208	257	201	284
Francisella tularensis subsp. holarctica (B)	BD07-537	Clinical isolate, Netherlands, 2007	223	290	195	10277	4130	5472	216	243	166	279	205	227	266	231	183	272
	LVS	Live vaccine strain, Russia	361	350	257	8755	4283	6902	352	330	304	348	283	273	302	250	292	269
	T8	Tick, Japan, 1957	243	171	252	9824	4484	4131	301	231	201	279	157	288	260	275	288	315
	T21 (FSC089)	Human blood, Norway, 1989	275	223	232	10039	4754	5920	273	302	267	260	273	307	178	287	252	324
	T20	Clinical isolate, France, 1994	281	179	248	11607	2949	7061	292	227	271	340	277	290	249	269	259	233
	T4	Vaccin strain SBL vaccin	307	261	243	8870	4391	5405	265	305	241	313	261	309	261	265	200	246
	T23	Clinical isolate, Sweden, 1998	256	253	274	9459	4340	5923	198	335	218	314	249	269	251	237	251	340
	T11	Clinical isolate, Sweden, 1984	281	261	290	9533	4381	7412	239	257	271	354	273	338	262	265	230	326
Francisella tularensis subsp. novicida	ATCC 15482	Water, USA, 1951	322	355	256	11193	3502	361	8550	10385	261	337	359	306	280	272	269	223
Yersinia pestis	Kenya 164	Biovar antiqua, Kenya, <1952	741	196	215	230	300	318	305	148	10956	6114	6542	10921	294	258	312	293
	Harbin	Biovar mediaevalis, China, <1948	208	360	307	251	310	287	251	257	13474	6691	10670	12511	253	295	315	261
	KIM3	Biovar mediaevalis, Kurdistan, 1968	201	213	219	216	218	270	212	244	15671	4254	203	12437	192	220	186	209
	Madagascar 34-94	Biovar orientalis, Madagascar	296	275	250	253	284	358	250	258	12674	6726	7636	11322	421	275	200	328
	Madagascar 73-93	Biovar orientalis, Madagascar	1719	248	264	198	305	271	206	241	7942	4731	5674	10459	215	208	194	191
	Nha Trang 63-115	Biovar orientalis, Vietnam	227	265	247	243	307	306	196	270	13390	6103	8320	228	280	183	262	287
	Hamburg 19	Biovar orientalis, Hamburg	213	254	190	259	304	252	264	233	13478	6490	268	12609	217	249	269	335
	EV 76	Biovar orientalis, Madagascar	325	267	258	258	235	328	232	179	13862	6392	7196	12942	236	284	286	269
	Peru 184	Peru	235	278	344	270	225	293	243	229	13900	7196	10442	12821	308	224	324	391
	NCTC 5923	Java, 1939	212	282	256	193	225	262	227	247	14965	4669	9889	13467	268	219	189	260
	NCTC 10029	Human bubonic plague, Nairobi, 1958	296	308	314	283	201	321	329	232	292	5361	7989	12550	307	297	255	263
	NCTC 10030	Human fatal bubonic plague, Nairobi, 1958	1506	281	274	292	283	329	241	290	330	4708	6834	11857	275	246	249	355
	NCTC 10330	Nairobi, 1963	237	311	267	211	329	342	265	284	262	5188	7450	12066	263	261	260	322
Coxiella burneti	Nine Mile	*Dermacentor andersoni* (tick), Montana, 1937	272	296	231	287	233	327	269	265	284	323	222	200	3587	2768	4562	276
	Priscilla	Goat cotyledon, USA, 1980	382	506	513	441	411	541	454	430	456	598	496	381	7924	7308	6741	970
	48	*Haemaphysalis punctata* (tick), Slovakia, 1967	369	342	443	517	464	489	449	442	374	433	482	470	9388	8610	8765	426
	S	Hepatitis, chronic Q-fever, USA, 1981	424	411	469	409	376	402	273	473	389	433	382	400	9317	8196	9716	437
	CbuG_Q212	Human hart valve, Nova Scotia, 1981	305	430	449	364	344	428	335	302	316	356	343	392	10119	8027	9439	341
	Henzerling	Human vaccine strain, Italy, 1945	357	323	319	345	364	355	410	312	348	430	372	403	9611	8547	9718	304
Bacillus atrophaeus	ATCC 9372		252	229	285	323	266	309	330	320	251	384	284	281	328	370	245	333
Bacillus cereus	ATCC 11778	NCIB Aberdeen, 1962	207	326	264	271	344	358	255	283	316	393	258	211	293	220	297	344
	BD03-229	Clinical isolate, Netherlands, 2003	319	301	314	339	270	294	301	269	261	392	334	271	316	293	287	355
	BD05-273	Clinical isolate, Netherlands, 2005	273	326	192	263	279	227	290	259	238	270	242	260	286	258	271	215
	WSBC 10530	Vomit from cooked rice, USA or UK, 1972	264	307	214	233	217	295	275	227	247	306	312	190	239	272	215	4218
	WSBC 10536	Indian rice dish, Germany/Pasau, 2001	237	289	278	263	301	313	291	317	231	440	209	297	391	293	226	5854
	WSBC 10583	Cooked rice, 1998	204	260	302	266	268	290	204	221	238	274	261	249	220	297	209	4983
	ATCC 10876	Contaminated bottle	224	250	219	207	250	264	177	218	219	356	299	224	249	244	274	260
	ATCC 7064	Blood	263	240	322	312	265	298	236	218	190	285	207	222	242	216	249	5313
	ATCC 14579		292	367	336	269	280	463	369	294	269	405	244	265	327	255	321	333
Bacillus coagulans		Purchased at Raven Labs, USA	345	286	263	1035	300	314	301	237	267	293	226	331	383	262	270	5161
Bacillus licheniformis	ATCC 9945	Flour, USA	222	216	273	243	252	356	261	360	270	299	259	267	360	318	249	310
Bacillus megaterium	ATCC 8245		321	281	292	306	284	430	284	256	226	442	292	308	390	290	230	5779
	ATCC 14581	Edinburgh, 1963	259	253	244	226	241	327	230	256	250	309	218	244	297	232	257	215
Bacillus mycoides	ATCC 6462		282	318	237	267	232	310	212	270	248	283	213	306	279	287	318	2659
Bacillus pumilus	ATCC 27142		292	193	200	231	276	311	254	220	257	312	248	297	291	181	280	217
Bacillus subtilis	ATCC 6633		822	407	276	243	315	431	233	294	285	330	306	303	408	348	326	287
Bacillus thuringiensis	ATCC 29730	var. galleriae Heimpel	271	287	207	267	235	402	191	257	235	347	312	274	291	202	270	2315
	ATCC 10792	var. berliner, Mediterranean flour moth	251	310	200	282	220	302	263	330	221	313	222	274	359	255	246	4287
	BD07-271	var. kurstaki	251	310	200	282	220	302	263	330	221	313	222	274	359	255	246	4287
	BD07-272	var. aizawai	251	310	200	282	220	302	263	330	221	313	222	274	359	255	246	4287
	BD07-273	var. galleriae	251	310	200	282	220	302	263	330	221	313	222	274	359	255	246	4287
Burkholderia mallei	NCTC 10229	Bird, Budapest	220	251	239	225	205	253	221	251	182	248	248	230	314	261	199	241
	NCTC 10230		220	251	239	225	205	253	221	251	182	248	248	230	314	261	199	241
Burkholderia pseudomallei	NCTC 10274	Blood of fatal human case, Kuala Lumpur, 1961	193	237	216	288	310	305	234	205	240	339	292	246	363	297	231	190
	NCTC 10276	Human, Liverpool, 1962	193	237	216	288	310	305	234	205	240	339	292	246	363	297	231	190
Clostridium bifermentans	PW14		272	310	214	234	232	302	261	320	210	469	311	234	303	262	283	273
Enterobacter cloacae	NCTC 13168		331	281	322	276	264	369	302	186	330	317	316	268	394	272	267	323
Escherichia coli	ATCC 25922	Clinical isolate, 1946	323	348	308	311	268	376	241	273	284	390	276	307	409	349	247	256
Francisella philomiragia	CCUG 13404	Clinical isolate, Switzerland, 1979	263	316	264	299	2165	330	278	248	252	291	266	283	301	165	277	208
	ATCC 25015	Moribund muskrat, Utah USA	217	205	290	251	262	229	298	217	265	278	273	309	287	228	213	240
Legionella anisa	ATCC 35292	Tap water, Los Angeles, 1981	279	305	218	193	189	358	271	228	234	306	197	230	323	234	219	199
Legionella pneumophila serotype 1	ATCC 33152	Human lung, 1977	185	280	227	447	220	266	264	286	199	255	256	228	301	236	212	247
Pantoea agglomerans	BD94-368	Clinical isolate, 1994	223	257	243	319	318	263	217	276	251	311	256	242	264	277	246	234
Pseudomonas aeruginosa	ATCC 15442	Animal room water bottle	240	221	212	258	238	420	276	236	258	302	263	251	274	252	275	239
	ATCC 27853	Blood culture, 1969	240	246	273	255	358	304	282	335	282	346	294	297	270	272	259	253
Salmonella enterica subsp. Enterica	ATCC 14028	Serovar Typhimurium. Bovine septicaemic liver, 1987	263	275	349	260	253	327	308	325	246	314	281	335	413	331	289	294
	ATCC 13076	Serovar Enteritidis	196	265	277	221	341	361	213	255	271	311	252	241	349	325	259	196
Yersinia enterocolitica	BD94-26	Clinical isolate, 1994	257	200	232	281	212	317	257	851	215	273	250	248	319	262	242	266
	BD93-563	Clinical isolate, 1993	257	200	232	281	212	317	257	851	215	273	250	248	319	262	242	266
	ATCC 9610	Human tissue, France, 1932	257	200	232	281	212	317	257	851	215	273	250	248	319	262	242	266
Yersinia frederiksenii	BD89-1545	Clinical isolate, 1998	254	209	186	262	184	308	256	243	256	361	276	219	253	249	228	362
Yersinia pseudotuberculosis	BD90-58	Clinical isolate, 1990	463	213	244	206	164	315	153	225	190	240	286	280	208	205	205	179
	BD90-89	Clinical isolate, 1990	463	213	244	206	164	315	153	225	190	240	286	280	208	205	205	179
	BD90-101	Clinical isolate, 1990	463	213	244	206	164	315	153	225	190	240	286	280	208	205	205	179
Bos taurus	469	Tissue experimental animal, Netherlands, 2009	206	217	212	216	288	352	237	263	239	368	235	225	312	296	328	260
Cerastoderma edule (common cockle foot)		Tissue, Netherlands, 2010	811	406	397	454	322	531	398	454	408	457	791	399	397	446	355	366
Chrysops relictus	I	Tissue, Netherlands, 2009	299	270	282	252	242	305	231	226	245	326	246	233	303	229	234	219
Ensis directus (razor clams foot)		Tissue, Netherlands, 2010	253	239	234	249	247	285	253	170	211	346	256	221	213	268	263	287
Felis catus	35	Blood, Netherlands, 2010	251	352	362	270	389	398	254	237	362	381	208	293	364	298	256	368
Homo sapiens	Volunteer 8	Blood, Netherlands, 2009	198	312	246	233	280	332	272	329	304	398	310	203	359	250	274	285
	Volunteer 10	Blood, Netherlands, 2009	260	193	230	232	259	283	281	378	447	301	240	213	356	297	312	350
Klebsiella pneumoniae	BD05-258	Clinical isolate, 2005	235	294	245	269	212	326	195	188	233	305	288	262	239	240	267	285
Mus musculus		Tissue, Netherlands, 2009	239	256	245	279	210	511	248	198	217	274	310	217	278	224	271	245
Mytilus edulis (mussel foot)		Tissue, Netherlands, 2010	242	252	173	239	181	365	236	269	260	315	275	229	253	211	171	237
Ovis aries	Twello 67	Tissue slaughterhouse, Netherlands, 2009	233	178	222	270	192	267	175	175	272	204	199	186	259	199	337	257
Rattus norvegicus	8604	Tissue, Netherlands, 2009	232	242	283	234	246	232	240	196	186	284	257	251	334	201	267	872
	8605	Tissue, Netherlands, 2009	232	242	283	234	246	232	240	196	186	284	257	251	334	201	267	872
Rattus rattus	8403	Tissue, Netherlands, 2009	271	254	275	237	264	291	245	262	199	338	10172	226	327	792	196	251
	8404	Tissue, Netherlands, 2009	271	254	275	237	264	291	245	262	199	338	10172	226	327	792	196	251
